# Adsorption structure of dimethyl ether on silicalite-1 zeolite determined using single-crystal X-ray diffraction

**DOI:** 10.1107/S2052520614015911

**Published:** 2014-10-01

**Authors:** Shinjiro Fujiyama, Shintaro Seino, Natsumi Kamiya, Koji Nishi, Yoshinobu Yokomori

**Affiliations:** a Department of Applied Chemistry, National Defense Academy, Hashirimizu, Yokosuka, Kanagawa 239-8686, Japan

**Keywords:** adsorption structure, MFI-type zeolite, silicalite-1, dimethyl ether, single-crystal structure analysis

## Abstract

The adsorption structures of dimethyl ether (DME) on silicalite-1 zeolite (MFI-type) are determined using single-crystal X-ray diffraction. The structure of low-loaded DME-silicalite-1 indicates that all DME molecules are located in the sinusoidal channel, which is the most stable sorption site based on the van der Waals interaction between DME and the framework. The configuration of guest molecules (linear or bent) plays an important role in determining where the stable sorption site is in the pore system of MFI-type zeolites. Bent molecules favor the sinusoidal channel, while linear molecules favor the straight channel. The contribution of DME–DME interactions is considerable in the high-loaded DME-silicalite-1 structure.

## Abstract 2

The most stable sorption site of dimethyl ether on silicalite-1 is the sinusoidal channel. The configuration of guest molecules (linear or bent) plays an important role in determining where the stable sorption site is situated.

## 1. Introduction   

Microporous materials such as zeolites, metal–organic frameworks and carbon nanomaterials are among the most important gas adsorbents. Gas molecules are physisorbed stably in micropores even around room temperature as a result of van der Waals interactions with the surrounding pore walls. The optimization of applications such as gas separation, storage and condensation requires knowledge of the effects of the pore structure on the adsorption behavior. Among the many potential microporous materials, zeolites are one of the most promising because of their high thermal, mechanical and chemical stability. The adsorption properties of various zeolites have been widely investigated. Above all, MFI-type zeolites, *e.g.* ZSM-5 and silicalite-1, have attracted much interest due to their two kinds of unique channels, a straight channel and a sinusoidal channel. Thermodynamic measurements were carried out on the adsorption of hydrocarbons (Richards & Rees, 1987 ▶; Shen & Rees, 1991 ▶; Choudhary & Mayadevi, 1996 ▶; Millot *et al.*, 1998 ▶, 1999 ▶; Sun *et al.*, 1996 ▶, 1998 ▶) and various other gases (Yamazaki *et al.*, 1993 ▶; Wirawan & Creaser, 2006 ▶; Pope, 1993 ▶; Golden & Sircar, 1994 ▶; Ahunbay *et al.*, 2008 ▶; Zhang *et al.*, 2012 ▶). The mobility of guest molecules in the pore system was studied using NMR spectroscopy (Shen *et al.*, 1990 ▶; Kolokolov *et al.*, 2010 ▶; Nishchenko *et al.*, 2012 ▶), and computational studies were also conducted to reveal the diffusion behavior (Makrodimitris *et al.*, 2001 ▶; Krishna *et al.*, 2006 ▶). A wide range of data has been reported, but very few actual adsorption structures have been reported except for aromatic molecules (van Koningsveld *et al.*, 1989 ▶; van Koningsveld, Jansen & Man, 1996 ▶, van Koningsveld, Jansen & van Bekkum, 1996 ▶; van Koningsveld & Jansen, 1996 ▶; van Koningsveld & Koegler, 1997 ▶; Nishi *et al.*, 2005 ▶; Kamiya *et al.*, 2011 ▶, 2013 ▶) and CO_2_ (Fujiyama *et al.*, 2013 ▶, 2014*a* ▶,*b* ▶). Determining the adsorption structures is important to understand the adsorption properties. Adsorption structures contain valuable information, such as stable sorption sites, the location and orientation of guest molecules, and guest–framework distances.

As mentioned above, many adsorption structures for aromatic molecules on MFI-type zeolites have been determined. These indicate that the intersection is the most stable sorption site based on van der Waals interactions between the guest molecules and the framework. This means that bulky aromatic molecules favor large intersections as the sorption site, rather than the narrow channels. Recently we revealed the adsorption process of CO_2_ on silicalite-1 using single-crystal X-ray structural analysis (Fujiyama *et al.*, 2014*b* ▶). CO_2_ molecules initially adsorb not at the intersection but in the straight channel through a CO_2_ framework interaction. It is reasonable that small molecules such as CO_2_ would favor the narrow channels rather than the large intersections. This can also be explained using an integrated Lennard–Jones potential model by treating the channels as simple cylinders (Tjatjopoulos *et al.*, 1988 ▶). However, this model cannot explain why CO_2_ molecules favor the straight channel rather than the sinusoidal channel. The difference in the pore sizes of the channels is too small to use the simple cylindrical potential model. The pore sizes of the channels are roughly the same, but the precise structures of the channels are quite different. This difference plays an important role and thus it should be considered in any discussion of the adsorption behavior in the channels. The channels are composed of two ten-membered rings (ten Si atoms and ten O atoms) with six O atoms connecting them. As discussed elsewhere (Fujiyama *et al.*, 2013 ▶), the ten-membered rings of the straight channel are parallel while those of the sinusoidal channel are angled. Considering the structural difference between the channels, the configuration of guest molecules (linear or bent) should be a key factor in determining which channel is the stable sorption site. The adsorption of guest molecules on silicalite-1 is based on the van der Waals interaction. The distances between the atoms of the guest molecule and the framework are important factors in determining the stability of a sorption site. A guest molecule on a stable sorption site favors those distances that minimize the van der Waals interaction potentials. The linear configuration of the CO_2_ molecule (O—C—O = 180°) may be compatible with the straight channel incorporating parallel ten-membered rings.

In this study we conducted a structural analysis of silicalite-1 loaded with dimethyl ether as a basic and simple example of guest molecules with a bent configuration (C—O—C = 111.7°). The direct comparison of the results with those of CO_2_, which is also a basic and simple example of chain molecules with linear configuration, is permitted because they have similar chain lengths and bulkiness. Adsorption structures were determined for low and high loading to discuss the guest–framework and guest–guest interactions separately.

## 2. Experimental   

### 2.1. Preparation of low- and high-loaded DME-silicalite-1   

Silicalite-1 crystals were prepared as reported elsewhere (Kamiya *et al.*, 2008 ▶, 2011 ▶). EDX analysis confirmed that the composition of the crystals was SiO_2_ with no Al or other cation species. Crystals selected for X-ray structural analysis were pressed by applying a mass of 2.0 g along the crystallographic *c* axis, while raising the temperature from ambient to 473 K and cooling back to ambient. This heating and cooling cycle was repeated three times for each specimen (Kamiya *et al.*, 2011 ▶). The crystal was exposed to DME gas at 90 kPa, 298 K for 12 h (low-loaded) or 7 d (high-loaded) in a closed vacuum instrument (Bell jar-type vacuum oven BV-001, Sibata Scientific Technology Ltd).

### 2.2. Structure analysis of DME-silicalite-1   

**Figure 1:**
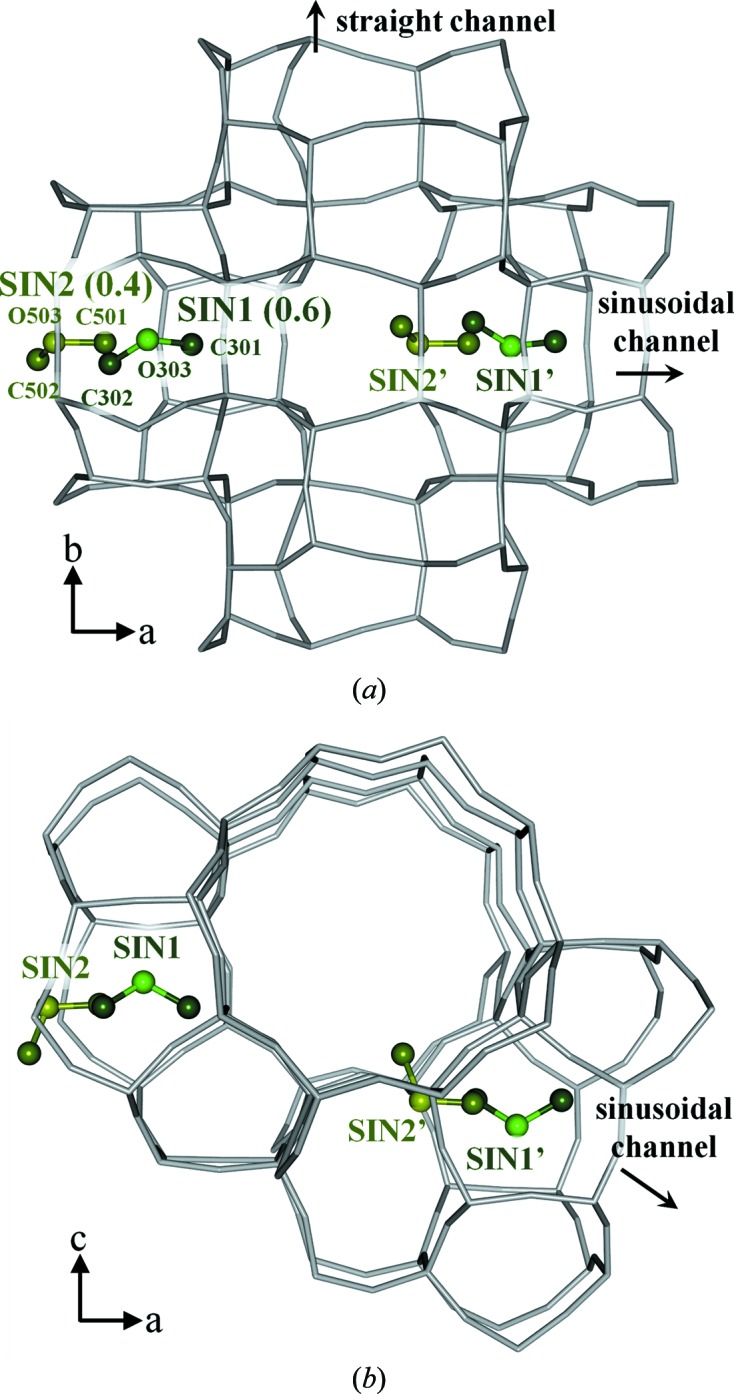
Packing of DME molecules in the low-loaded DME-silicalite-1 (*a*) along the *c* axis and (*b*) along the *b* axis, with the occupancy factors indicated in parentheses.

**Figure 2:**
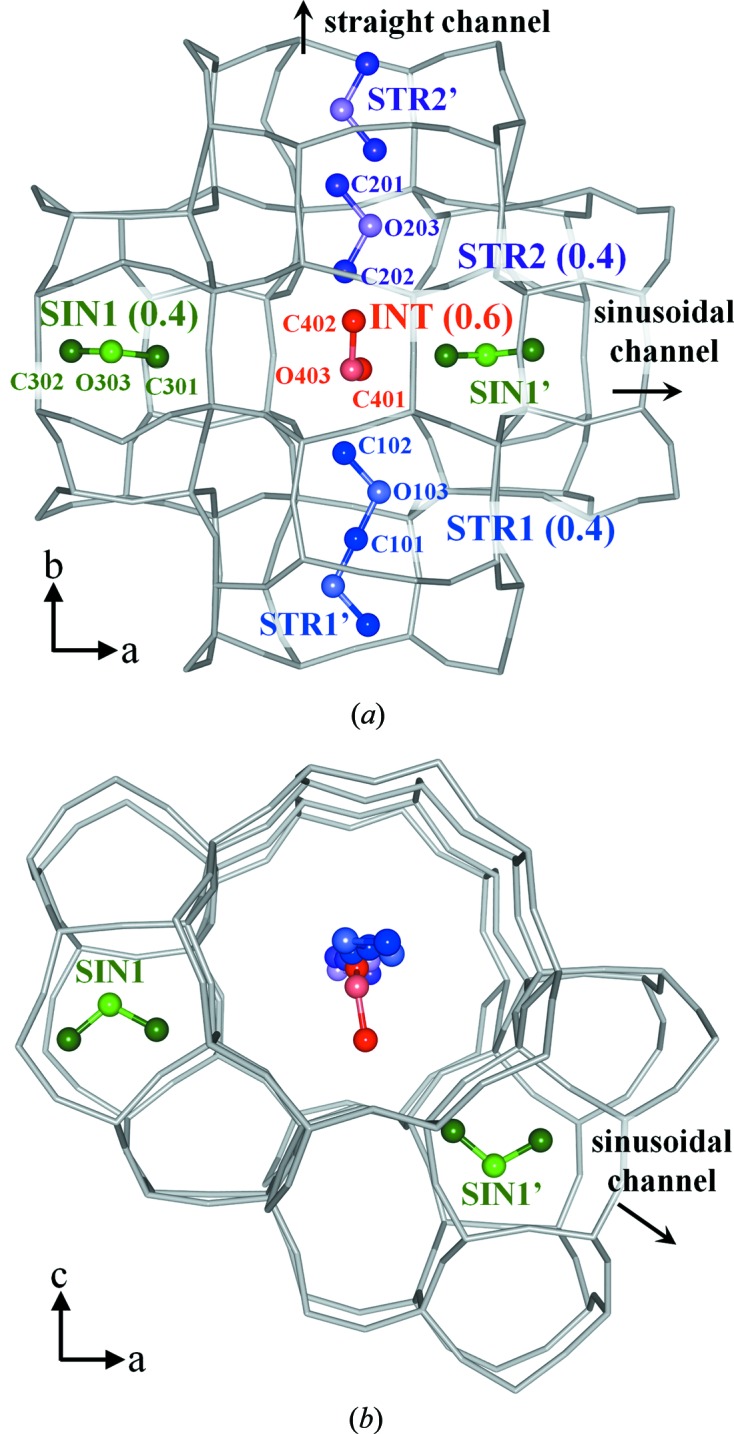
Packing of DME molecules in the high-loaded DME-silicalite-1 (*a*) along the *c* axis and (*b*) along the *b* axis, with the occupancy factors indicated in parentheses.

Single-crystal X-ray diffraction data was collected at room temperature using an APEX II X-ray diffractometer (Bruker AXS) with a CCD detector, Mo *K*α radiation, and a graphite monochromator. The collected reflections were corrected for Lorentz polarization factors and the absorption effect. Structural analysis was conducted in the monoclinic twin in *P*2_1_/*n*.1.1 as described in the report (Fujiyama *et al.*, 2014*a* ▶). The structure was solved using a direct method, and difference-Fourier synthesis was used for the remaining atoms (*SHELXTL*; Sheldrick, 2008 ▶). Refinement was performed on *F* ^2^ and Σ*w*(*F*_o_^2^ − *F*_c_^2^)^2^ was minimized; *w* = 1/[σ^2^(*F*_o_^2^) + (*aP*)^2^ + *bP*], where *P* = (*F*_o_^2^ + 2*F*_c_^2^)/3, and *a* and b are the weight parameters. Anisotropic displacement parameters were used and no restraints were introduced on the framework atoms. Isotropic displacement parameters were used on the DME atoms and the structures were constrained as rigid groups (C—O = 1.41 Å, C—O—C = 111.7°). The unstable displacement parameters of DME atoms were restrained. In the refinement of the high-loaded DME-silicalite-1, the sums of the occupancy factors of two pairs of disordered DMEs (STR2–INT and SIN1–INT in Fig. 2) were restrained to be 1.0. The full experimental details are given in Table 1 ▶ and the structures of DME-silicalite-1 are shown in Figs. 1 ▶ and 2 ▶. The structures were drawn using the software *VESTA* (Momma & Izumi, 2008 ▶).

### 2.3. Thermogravimetric analysis   

**Figure 3:**
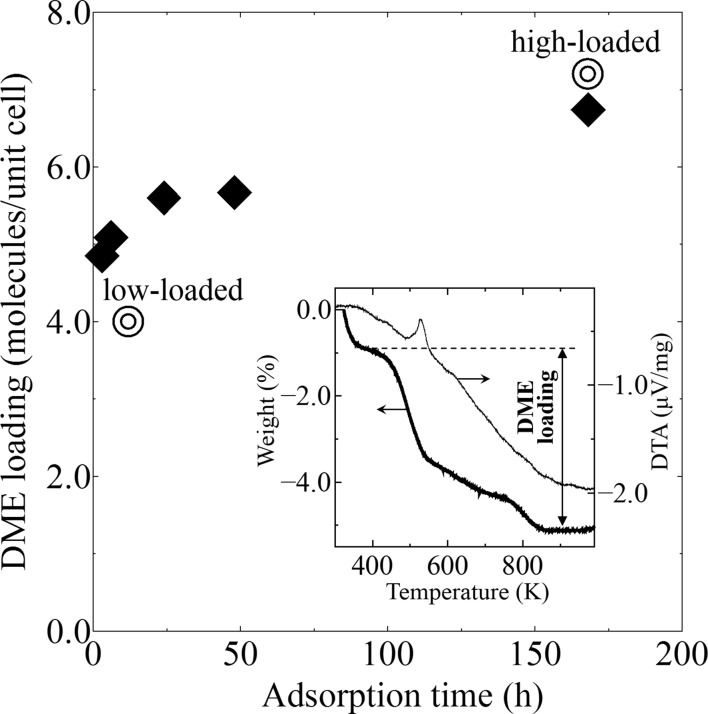
DME loading on silicalite-1 measured by thermogravimetric analysis (diamonds), along with the DME amounts calculated using the occupancy factors of XRD analysis (double circles). Insert: TG–DTA curves of DME-silicalite-1 (adsorption time 7 d).

The amount of DME loading on silicalite-1 was measured thermogravimetrically to validate the occupancy factors of the DME-silicalite-1 structures. Silicalite-1 crystals weighing 10 mg each were exposed to DME gas at 90 kPa, 298 K, in a closed vacuum instrument (Bell jar type vacuum oven BV-001, Shibata Science Co.). The adsorption times used were 3, 6, 24, 48 h and 7 d. The resultant crystals were placed in a Bruker TG–DTA (thermogravimetry–differential thermal analysis) 2000SA sample holder and heated at 2 K min^−1^ in flowing air. The weight loss of the crystals was measured up to 1000 K. The plot for each sample is shown in Fig. 3 ▶. The TG–DTA curves of the adsorption time (7 d) is inserted as a typical example.

## 3. Results   

### 3.1. Packing of DME in silicalite-1   

The packing of DME in low-loaded DME-silicalite-1 is shown in Fig. 1 ▶ with the occupancy factors in parenthesis. Two independent DME sorption sites are observed in the sinusoidal channel. SIN1–SIN1′ and SIN2–SIN2′ are related to the screw axis 2_1_ along the *a* axis. SIN1 is located between the two ten-membered rings and SIN2 is located in the middle of one ring. The sum of the occupancy factors is 1.0, which means the sinusoidal channel is fully occupied. The amount of DME calculated using the occupancy factors is 4.0 molecules/u.c. Fig. 2 ▶ shows the packing of DME in the high-loaded DME-silicalite-1. Four independent sorption sites are observed. SIN1 is also observed in the high-loaded structure. STR1–STR1′ and STR2–STR2′ are related to the symmetric center in the middle of the straight channel, while SIN1–SIN1′ are related to the screw axis 2_1_ along the *a* axis. C101 of the STR1 is at the symmetric inversion center. The amount of DME calculated using the occupancy factors is 7.3 molecules/u.c. The DME loading measured by thermogravimetric analysis is shown in Fig. 3 ▶ along with the amount of DME calculated using the occupancy factors of the XRD results. The results agree and thus validate the low- and high-loaded structures. The first weight loss from room temperature to about 400 K in the TG curve is mainly due to DME molecules adsorbed out of the pores. The DME-to-framework internuclear distances in low- and high-loaded DME-silicalite-1 are listed in Tables 2 ▶ and 3 ▶. The numbering of the framework atoms is identical to the single-crystal structure in *P*2_1_/*n*.1.1 (van Koningsveld *et al.*, 1990 ▶).

### 3.2. Framework geometry of DME-silicalite-1   

**Figure 4:**
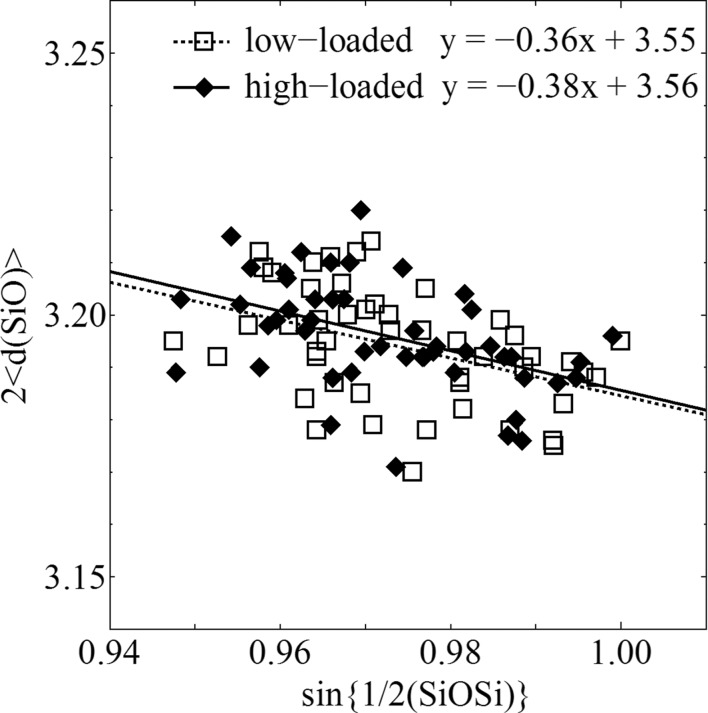
Scatter diagram of 〈*d*(SiO)〉 as a function of the Si—O—Si angle, with the equations of the regression lines.

The bond lengths and angles in the framework geometry and the diagonal O—O internuclear distances in the ten-membered rings of the channels are listed in Tables 4 ▶ and 5 ▶. The l/s value in Table 5 ▶ is the longest distance divided by the shortest distance, which indicates the local strain of the channel. The scatter diagram of 〈*d*(SiO)〉 (the average of the two Si—O distances of each Si—O—Si bridge) as a function of the Si—O—Si angle is shown in Fig. 4 ▶. The absolute value of the slope of the regression line indicates the strain in the whole framework geometry, and the larger values can be attributed to a more stressed structure.

## 4. Discussion   

### 4.1. Sorption sites based on DME–framework interaction   

**Figure 5:**
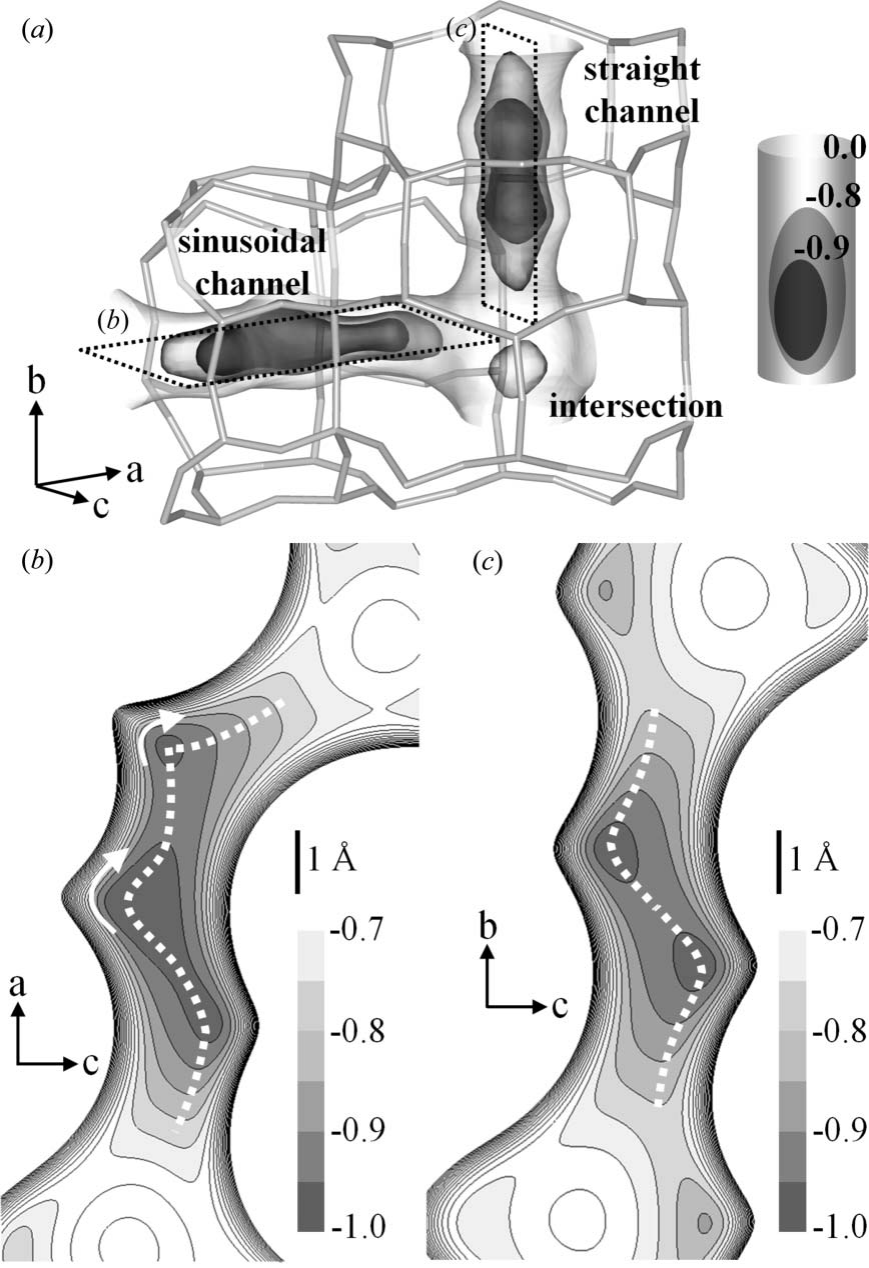
Profile of normalized *U*_atom–framework_. (*a*) Overview of the profile and the framework structure of the channels. Isosurfaces are at −0.9, −0.8 and 0.0. Contour maps of the (*b*) sinusoidal and (*c*) straight channels. The contour lines are −0.95 to 0.00 in increments of 0.05. Minimum potential paths of each channel are indicated by dashed lines.

**Figure 6:**
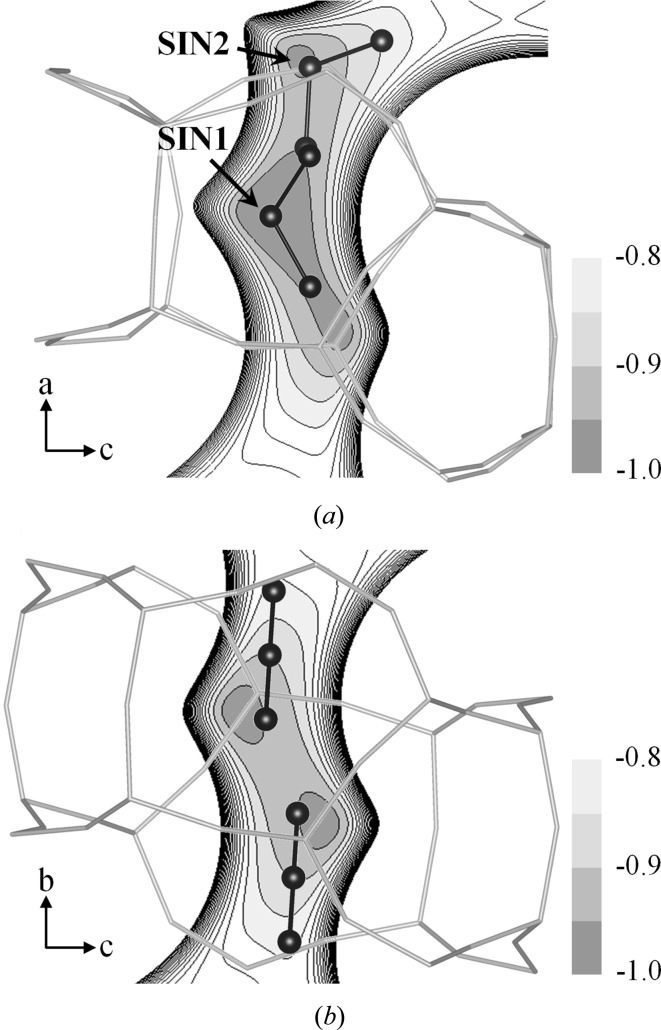
Structures of guest molecules with *U*_atom–framework_ maps. (*a*) SIN1 and SIN2 of low-loaded DME. (*b*) STR2 of low-loaded CO_2_ (Fujiyama *et al.*, 2014*b* ▶).

The structure of the low-loaded DME-silicalite-1 clearly indicates that the sinusoidal channel is the most stable sorption site of DME based on the DME–framework interaction. The DME–DME interaction in the low-loaded structure is negligibly small because the DME molecules are located separately. The shortest distance between neighboring DMEs is over 6.0 Å (SIN1 to SIN2), which is too long for the DME–DME interaction to work. As expected, DME does not adsorb at the intersection initially. As mentioned in §1, it is not surprising that small molecules such as DME favor the narrow channels rather than the large intersection. The precise structures of the framework atoms of the channels should be considered in order to explain why DME molecules favor the sinusoidal channel rather than the straight channel. The Lennard–Jones potential model can be used to estimate the guest–framework interaction by taking into account the precise structures of the channels. The guest–framework interaction potential exhibits additive properties for atoms of the guest molecule. For example, the experimental enthalpy variations of hydrocarbons increase linearly as a function of the carbon number. The increase is approximately 10 kJ mol^−1^ per additional CH_2_ group from butane to hexane (Richards & Rees, 1987 ▶). Thus the interaction potential between the guest molecule and the framework, *U*_molecule–framework_, can be considered as the sum of the interaction potentials of all atoms of the guest molecule as follows
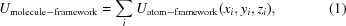
where *U*_atom–framework_ (*x_i_, y_i_, z_i_*) is the interaction potential of atom *i* at the position (*x_i_, y_i_, z_i_*) in the coordinate space; *i* = 1, 2, 3 in the case of a triatomic molecule. The profile of *U*_atom–framework_ (*x*, *y*, *z*) in the channels helps to evaluate the stability of the sorption sites from the perspective of the configuration of the guest molecules. The atoms of a molecule on a stable sorption site would be located at positions where the *U*_atom–framework_ (*x*, *y*, *z*) is low. The *U*_atom–framework_ (*x*, *y*, *z*) can be expressed approximately as the sum of the Lennard–Jones potential between the atom at (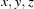) and the overall framework atoms. For simplicity, *U*_atom–framework_ (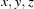) is calculated under the following assumptions. The Si atoms of the framework are excluded from the calculation and 426 framework O atoms around the channels are counted. The van der Waals radius of the guest atom, which represents the radii of common atoms such as carbon, nitrogen and oxygen, is taken to be approximately 1.70 Å. Then, *U*_atom–framework_ (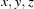) is given by
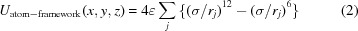


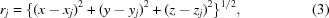
where (*x_j_*, *y_j_*, *z_j_*) is the position of the framework atom *j*, σ is the separation at which the potential becomes zero, and 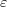 is the depth of the potential well. The factor 4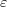; can be canceled by normalizing *U*_atom–framework_ (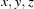). The value of σ is given by the relationship
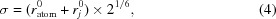
where *r* ^0^_atom_ and *r* ^0^ *_j_* are the van der Waals radii of the atoms of the guest molecule and the framework. *r* ^0^_atom_ is 1.70 Å, and *r* ^0^ *_j_* is 1.52 Å for any *j*, which is the van der Waals radius of O. The isosurfaces of normalized *U*_atom–framework_ (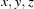) at 0.0, −0.8 and −0.9 are shown in Fig. 5 ▶(*a*). The isosurface at 0.0 runs through the entire channel system. There are deep potential wells in the channels and a shallow local minimum is found in the area of the intersection. Figs. 5 ▶(*b*) and (*c*) show the details of the potential wells in the channels. Their depths in the sinusoidal channel and the straight channel are approximately the same, the difference being less than 2%. This stands to reason considering that their pore sizes are roughly the same. However, the configuration of the potential wells is clearly different. As can be seen in the minimum potential paths indicated by the dashed lines, the path of the sinusoidal channel is winding and that of the straight channel is linear. Thus, the sinusoidal and straight channels are more favorable for, respectively, bent and linear molecules to locate their atoms at stable positions. Fig. 6 ▶ shows the locations of the most stable sorption sites in low-loaded DME-silicalite-1 (this work) and low-loaded CO_2_-silicalite-1 (Fujiyama *et al.*, 2014*b* ▶) with the potential well maps. They are located around the potential wells as expected. The bent molecular chains of DME fit exactly along the bent potential wells. The location of CO_2_ does not coincide as perfectly with the potential well as that of DME, but CO_2_ is along the linear minimum potential path, with the O atom on the potential minimum side. The matching between the configurations of the guest molecule and the potential wells of the channels determines which channel is the stable sorption site for the guest molecule. Bent molecules favor the sinusoidal channel, while linear molecules favor the straight channel.

### 4.2. Adsorption process of DME on silicalite-1   

**Figure 7:**
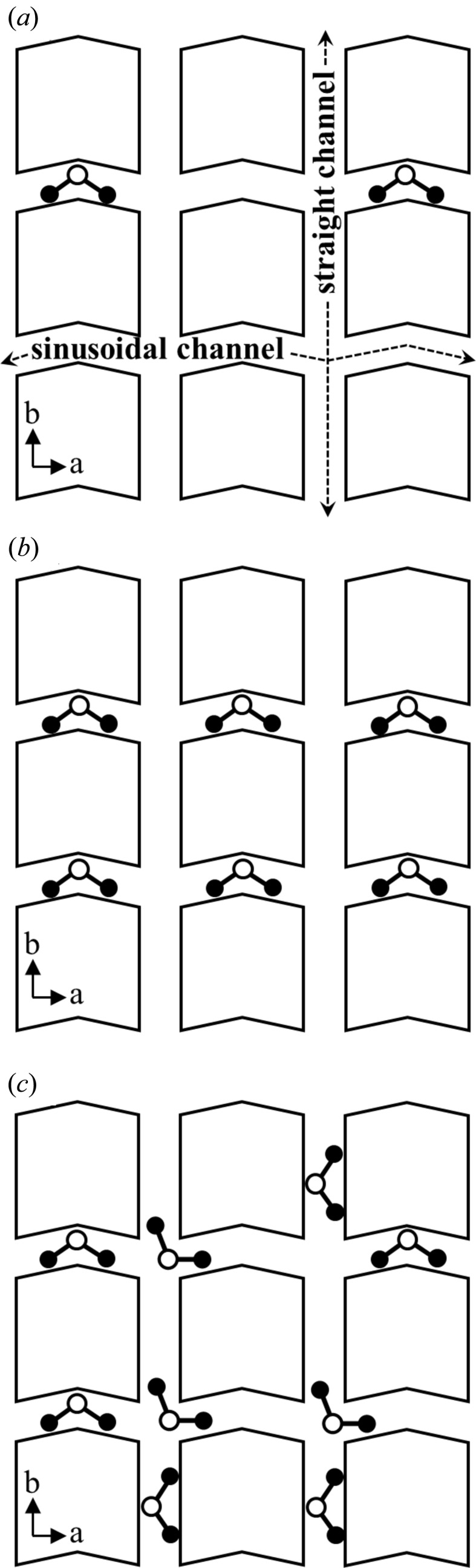
Adsorption process of DME on silicalite-1. (*a*) Every DME molecule is initially located in the sinusoidal channel. (*b*) The sinusoidal channel is fully occupied (low-loaded structure). (*c*) Under equilibrium conditions at 90 kPa, 298 K (high-loaded structure).

The DME molecules in the low-loaded structure undergo rearrangement in the high-loaded structure due to the DME–DME interaction. Fig. 7 ▶ illustrates the adsorption process of DME on silicalite-1. The initial adsorption behavior is governed by the DME–framework interaction. Up to a DME loading of 4 molecules/u.c., all DME molecules are located in the sinusoidal channel as a result of the DME–framework interaction. The additional DME molecules adsorb in the straight channel and/or at the intersection where the loading is over 4 molecules/u.c. Then the DME–DME interaction arises and some of the DME molecules in the sinusoidal channel move to the straight channel or the intersection. In the high-loaded DME-silicalite-1 structure, a considerable amount of DME is located at the intersection. The occupancy factor at the intersection (0.6) is larger than in the channels (0.4). The large intersection is less stable than the narrow channels for small molecules such as DME according to the DME–framework interaction. As listed in Table 3 ▶, the DME–framework distances of INT are larger than 4.0 Å, which is too long to minimize the DME–framework interaction potentials (see Table 2 ▶). Thus, DME molecules at INT are stabilized by the DME–DME interaction, which comprises a dipole–dipole interaction as well as a van der Waals interaction. The orientation of DME molecules at the large intersection has a high degree of freedom, and thus the dipole–dipole interactions in the high-loaded structure are optimized. The adsorption behavior of CO_2_ on silicalite-1 shows the same tendency. A large number of CO_2_ molecules are located at the large intersection stabilized by the CO_2_–CO_2_ interaction in the high-loaded structure.

### 4.3. Strain in silicalite-1 framework loaded with DME   

The results relevant to the entire framework geometry in Table 4 ▶ and Fig. 4 ▶ and the local strain in the channels in Table 5 ▶ are identical to those for monoclinic single crystals with no guest molecules in their pores (van Koningsveld *et al.*, 1990 ▶; Kamiya *et al.*, 2010 ▶). Unlike bulky aromatic compounds, DME molecules and CO_2_ are too small to exert any influence on the framework geometry. The framework geometry loaded with aromatic compounds is orthorhombic, and the absolute values of the slopes of the regression lines are around 0.5. The channels are also distorted with the bulky aromatic molecules in them and their l/s values are over 1.2.

## 5. Conclusion   

The structures of low- and high-loaded DME-silicalite-1 were determined. The sinusoidal channel is found to be the most stable sorption site for DME molecules. Up to a DME loading of 4 molecules/u.c., all DME molecules are located in the sinusoidal channel as a result of the DME–framework interaction. The configuration of the guest molecules (linear or bent) plays an important role in determining which channel is the most stable sorption site based on the guest–framework interaction. Linear molecules favor the straight channel, while bent molecules favor the sinusoidal channel. In the high-loaded structure, a large amount of DME is located at the intersection owing to the DME–DME interaction.

Recently, we have reported the adsorption structures of C_4_–C_6_ hydrocarbons (Fujiyama, Seino *et al.*, 2014 ▶). Linear 2-butyne prefers the straight channel, and bent *n*-butane prefers the sinusoidal channel as expected. Further investigations about other chain molecules are needed to reveal the adsorption behavior in the MFI-type zeolites.

## Supplementary Material

Crystal structure: contains datablock(s) I, II. DOI: 10.1107/S2052520614015911/bp5065sup1.cif

Structure factors: contains datablock(s) I. DOI: 10.1107/S2052520614015911/bp5065Isup2.hkl

Structure factors: contains datablock(s) II. DOI: 10.1107/S2052520614015911/bp5065IIsup3.hkl

Comments on the checkcif (alerts level A and B). DOI: 10.1107/S2052520614015911/bp5065sup4.pdf

CCDC reference: 1012728

## Figures and Tables

**Table 1:** Crystal data and refinement details

	Low-loaded	High-loaded
Crystal data		
Chemical formula	Si_24_O_48_·0.96C_2_O	Si_24_O_48_·1.82C_2_O
*M*_r_	1480	1515
Crystal system, space group	Monoclinic, *P*2_1_/*n*.1.1	Monoclinic, *P*2_1_/*n*.1.1
Temperature (K)	296	296
*a*, *b*, *c* (Å)	20.186 (15), 19.990 (14), 13.435 (10)	20.169 (14), 19.951 (14), 13.427 (10)
α (°)	90.012 (13)	90.012 (13)
*V* (Å^3^)	5421 (7)	5421 (7)
*Z*	4	4
Radiation type	Mo *K*α	Mo *K*α
μ (mm^−1^)	0.67	0.67
Crystal size (mm)	0.16 × 0.11 × 0.08	0.14 × 0.12 × 0.08

Data collection		
Diffractometer	Bruker P4	Bruker P4
Absorption correction	Analytical	Analytical
*T*_min_, *T*_max_	0.899, 0.950	0.912, 0.951
No. of measured, independent and observed [*I* > 2σ(*I*)] reflections	63 627, 13 249, 6100	63 444, 13 209, 5947
*R*_int_	0.091	0.115
(sin θ/λ)_max_ (Å^−1^)	0.676	0.676

Refinement		
*R*[*F* ^2^ > 2σ(*F* ^2^)], *wR*(*F* ^2^), *S*	0.049, 0.124, 0.85	0.054, 0.124, 0.83
No. of reflections	13 249	13 209
No. of parameters	664	669
No. of restraints	4	10
Δρ_max_, Δρ_min_ (e Å^−3^)	0.74, −0.60	0.63, −0.50

Computer programs: *XSCANS* (Siemens, 1996 ▶), *SHELXTL*, *SHELXS97*, *SHELXL97* (Sheldrick, 2008 ▶).

**Table 2:** DME-to-framework internuclear distances (Å) in the low-loaded DME-silicalite-1

SIN1 to framework		SIN2 to framework	
C301—O25	3.90	C501—O4	3.91
C302—O4	3.42	C502—O21	3.68
O303—O17	3.92	O503—O26	3.90

**Table 3:** DME-to-framework internuclear distances (Å) in the high-loaded DME-silicalite-1

STR1 to framework			
C101—O47	4.01	C201—O28	3.54
C102—O2	3.76	C202—O11	3.76
O103—O31	3.63	O203—O34	3.71
			
SIN1 to framework		INT to framework	
C301—O18	3.84	C401—O15	4.02
C302—O31	3.87	C402—O34	4.13
O303—O17	3.59	O403—Si9	4.81

**Table 4:** Bond lengths (Å) and angles (°) in the framework geometry

	Low-loaded	High-loaded
O—Si—O range (°)	106–112	107–113
Average O—Si—O (°)	109	109
		
Si—O range (Å)	1.56–1.62	1.56–1.62
Range of averages of Si—O/SiO_4_ (Å)	1.59–1.60	1.59–1.60
		
Si—O—Si range (°)	143–179	143–175
Range of averages of Si—O—Si/Si(OSi)_4_ (°)	149–163	148–161

**Table 5:** Diagonal O—O internuclear distances (Å) in ten-membered rings

	Low loaded	High loaded		Low loaded	High loaded
Straight channel					
O5—O11	8.08	8.14	O31—O37	8.31	8.39
O1—O20	8.43	8.47	O44—O46	8.40	8.46
O34— O28	8.05	7.95	O8—O2	8.27	8.24
O33—O27	8.32	8.26	O7—O1	8.24	8.14
O22—O21	8.11	8.12	O48—O47	8.07	8.05
l/s†	1.05	1.06	l/s†	1.04	1.05
					
Sinusoidal channel					
O15—O20	8.21	8.18	O18—O17	7.90	7.88
O1—O28	8.14	8.08	O5—O30	8.34	8.46
O2—O27	8.12	8.14	O4—O31	7.86	7.76
O46—O41	8.50	8.56	O43—O44	8.11	8.13
O24—O26	8.16	8.15	O23—O25	8.45	8.42
l/s†	1.05	1.06	l/s†	1.08	1.09

† Longest distance divided by the shortest distance.
