# Supplementary material for: Adsorption structure of dimethyl ether on silicalite-1 zeolite determined using single-crystal X-ray diffraction
Source: Acta Crystallogr B Struct Sci Cryst Eng Mater. 2014 Oct 1;70(Pt 5):856–63. doi: 10.1107/S2052520614015911 (PMC4184374; doi:10.1107/S2052520614015911)
Supplement: Supplementary file 4 [file b-70-00856-sup4.pdf]

Comments on the checkcif (alerts level A and B)

PLAT213_ALERT_2_B Atom O15 has ADP max/min Ratio ..... 4.1 oblate

The unordinary displacement parameters of framework oxygen atoms are due to the lack of crystalline order. The framework of the silicalite-1 undergoes many phase transitions with varying mechanical stress, temperature change or adsorption of guest compounds. This property suggests that the framework geometry of the silicalite-1 is very flexible, and it is a relatively soft crystal. The framework atoms are in disorder, and it is observed as the unordinary displacement parameters.

PLAT029_ALERT_3_B diffrn_measured_fraction_theta_full Low

This alert is generated due to the number of lost reflections at higher theta area. DME-silicalite-1 is in the twin phases and their lattices are so close that they can be treated as nearly coincident. The reflections in lower theta area are overlapped and those in higher theta area are non-overlapped. In this work, the overlapped reflections were measured and pseudo-merohedral twin refinement was conducted. The non-overlapped reflections in the higher theta area are not observed, so this method makes the completeness in this area slightly diminished. However, at least 94% overall completeness is acquired and the structural analysis successfully works.

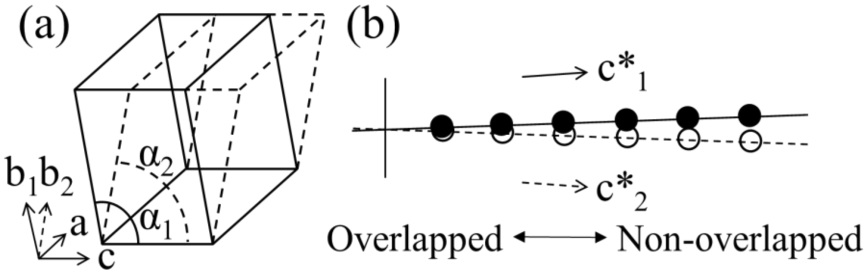

Figure (a) Monoclinic twin phases of silicalite-1, (b) Reflections along the *c** axis.

PLAT602_ALERT_2_A VERY LARGE Solvent Accessible VOID(S) in Structure.

Silicalite-1 is the porous substance, so the existence of “solvent accessible voids” is not a problem to be resolved.

The setting of cell parameters in the paper is unique axis *a* (β = γ = 90°, α > 90°). The cif and fcf files for the checkcif are transformed to be unique axis *b* (α = γ = 90°, β > 90°) to make the checkcif program work appropriately. The program seems to round the α (> 90°) in the cif file to 90° and out put the error “PLAT 910 (Test for CIF & FCF CELL Not Matching)”.
